# Dating violence among adolescents from a region of high social vulnerability*

**DOI:** 10.1590/1518-8345.5353.3499

**Published:** 2021-11-08

**Authors:** Ana Paula França de Oliveira, Silvia Mara Carvalho Silva, Ana Beatriz Campeiz, Wanderlei Abadio de Oliveira, Marta Angelica Iossi Silva, Diene Monique Carlos

**Affiliations:** 1 Universidade Federal de São Carlos, Departamento de Enfermagem, São Carlos, SP, Brazil.; 2 Scholarship holder at the Fundação de Amparo à Pesquisa do Estado de São Paulo (FAPESP), Brazil.; 3 Scholarship holder at the Coordenação de Aperfeiçoamento de Pessoal de Nível Superior (CAPES), Brazil.; 4 Universidade de São Paulo, Escola de Enfermagem de Ribeirão Preto, PAHO/WHO Collaborating Centre for Nursing Research Development, Ribeirão Preto, SP, Brazil.; 5 Scholarship holder at the Conselho Nacional de Desenvolvimento Científico e Tecnológico (CNPq), Brazil.; 6 Pontifícia Universidade Católica de Campinas, Campinas, SP, Brazil.

**Keywords:** Intimate Partner Violence, Adolescent, Gender-Based Violence, Vulnerable Populations, Social Vulnerability, Qualitative Research, Violencia de Pareja, Adolescente, Violencia de Género, Poblaciones Vulnerables, Vulnerabilidad Social, Investigación Cualitativa, Violência por Parceiro Íntimo, Adolescente, Violência de Gênero, Populações Vulneráveis, Vulnerabilidade Social, Pesquisa Qualitativa

## Abstract

**Objective:**

to know and analyze the perceptions of adolescents with high social vulnerability regarding the establishment of dating violence.

**Method:**

a qualitative research study carried out with 19 adolescents from a central municipality São Paulo, Brazil. Data collection took place by means of focus groups and field diaries, with the data being analyzed thematically.

**Results:**

two categories emerged: “A new female posture in a context of traditional gender norms” and “Violence in intimate relationships: the (non)perception of adolescents”. Traditional gender norms still occupy a significant place in the design of dating violence among adolescents. Such behaviors are more visible in these relationships, when commitment and exclusivity are seen as the main characteristics, authorizing possession and control. Jealousy emerges as the main trigger for violence and the technologies appear as contemporary resources to reinforce it.

**Conclusion:**

the need for early interventions with adolescents is reiterated, with a focus on actions that promote gender equality.

## Abstract (es)

**Objetivo:**

conocer y analizar las percepciones de adolescentes con alta vulnerabilidad social sobre la construcción de relaciones de pareja violentas.

**Método:**

investigación cualitativa realizada con 19 adolescentes de un municipio central de São Paulo, Brasil. La recolección de datos se llevó a cabo a través de grupos focales y un diario de campo, y los datos se analizaron temáticamente.

**Resultados:**

surgieron dos categorías: “Una nueva postura femenina en un contexto de normas tradicionales de género” y “Violencia en las relaciones íntimas: la (no) percepción de los adolescentes”. Las normas tradicionales de género todavía ocupan un lugar importante en la configuración de las relaciones entre los adolescentes. Tales comportamientos son más visibles en los noviazgos, cuando el compromiso y la exclusividad son las principales características, autorizando la posesión y el control. Los celos surgen como el principal detonante de la violencia y la tecnología aparece como recurso contemporáneo para reforzarla.

**Conclusión:**

se recalca que es necesario realizar intervenciones precoces con los adolescentes, centradas en acciones que promuevan la equidad de género.

## Abstract (pt)

**Objetivo:**

conhecer e analisar as percepções de adolescentes de alta vulnerabilidade social frente à construção de relações de intimidade violentas.

**Método:**

pesquisa qualitativa realizada junto a 19 adolescentes de um município central de São Paulo, Brasil. A coleta de dados se deu por meio de grupos focais e diário de campo, sendo os dados analisados tematicamente.

**Resultados:**

emergiram duas categorias “Uma nova postura feminina em um contexto de normas tradicionais de gênero” e “Violências nas relações íntimas: a (não) percepção dos adolescentes”. Normas tradicionais de gênero ainda ocupam um lugar significativo no delineamento de relações entre adolescentes. Tais condutas são mais visíveis em relações de namoro, quando o compromisso e a exclusividade são tidos como principais características, autorizando a posse e o controle. O ciúme emerge como principal disparador de violência e as tecnologias aparecem como recursos contemporâneos para reforçá-la.

**Conclusão:**

reitera-se a necessidade de intervenções precoces junto a adolescentes, com foco em ações promotoras de equidade de gênero.

## Introduction

In recent years, a number of studies have denoted the importance of violence in intimate relationships among adolescents^(1-4)^. Such relevance is due to some factors, including the early establishment of unhealthy intimate relationships in people’s lives^(5)^, as well as socio-cultural constructions on the gender relations.

Several terms have been used in the literature to conceptualize this violence among adolescents, and the term has been used internationally as teen dating violence (TDV). It is considered as physical, sexual, psychological or emotional violence in a dating relationship, including persecution. It is important to highlight that such violence can occur in person or by electronic means, as well as among casual or formal intimate partners^(5)^.

TDV is a common phenomenon that cuts across cultures, races and ethnicities. A descriptive cross-sectional study whose participants were 403 adolescents between 14 and 19 years old, who reported having perpetrated some type of violence in their affective-sexual relationships during adolescence (62.4% female), high school students from public (64.5%), private (18.2%) and professional training (17.3%) schools, from the Metropolitan Region of Porto Alegre, Brazil^(6)^. A meta-analysis with data from 46 underdeveloped or developing countries indicated that young and rural women were more exposed to intimate partner violence^(7)^. A study with 930 pregnant black-skinned and Latino adolescents found that 38% experienced TDV in the last trimester of pregnancy^(8)^. A study conducted in 27 countries in sub-Saharan Africa found that a median of 25.2% of adolescents and young women reported TDV, reaching 43.3% in some countries^(9)^.

TDV has consequences for the adolescents’ physical and mental health in the short- and long-term. A systematic literature review showed that it is associated with problems such as depression, anxiety, low self-esteem, alcohol and drug abuse and unprotected sex^(10)^. In addition to that, adolescents involved in dating abuse are more likely to be involved in violent relationships in the adult phase^(11)^.

Understanding violence and its consequent prevention and coping are challenging and complex tasks, since they are rooted in social, economic and cultural factors - such as sexist gender norms - which, in turn, exert an influence on the daily lives of communities and families, as well as on the way in which these relationships are experienced by the adolescents^(12)^. In this sense, for understanding violence, the World Health Organization (WHO) proposes an ecological model, which is based on the evidence that no single factor can explain the greater risk and vulnerability of some people or groups to interpersonal violence, while others are more protected from it. Thus, such phenomenon is understood as a result of the interaction of multiple factors at four levels: individual, relational, community, and social^(13)^.

Considering the ecological model, the perspective to contexts of social vulnerability stands out, being understood as those with absence of elements such as income, schooling, possibilities of insertion in the labor market, and access to goods and services^(14)^. The weaknesses of affective-relational bonds can also be included in such concept. Considering vulnerability and its determining factors, both in its ethical, political and technical aspects, circumventing the incidence of risks in the territories, it is indispensable for the subjects, based on their capabilities and empowerment, to be able to face these vulnerabilities imposed in their daily lives^(15)^.

Being in a context of social vulnerability can expose adolescents to situations of violence and has impacts at several levels. A recent literature review on the causes and consequences of TDV showed that poverty is identified as an important factor for the prevalence of TDV^(10)^. Diverse evidence even suggests that financial stress and low income also increase the risk for TDV^(16)^. Reducing these situations can diminish the possibilities for relational conflicts^(17)^. In addition to this aspect, it is important to mention that regions with high social vulnerability are more prone to gender inequalities in education, employment and income, mainly when supported by the patriarchal culture and the power relationship between men and women^(10)^.

Considering the relevance and impact of TDV, especially for those inserted in contexts of high social vulnerability, as well as the importance of understanding this phenomenon from the ecological model, the following research question emerged: What are the perceptions of adolescents with high social vulnerability regarding their intimate relationships, considering healthy and violent aspects?

Therefore, this study aimed at understanding and analyzing the perceptions of adolescents with high social vulnerability regarding the establishment of violent intimate relationships.

## Method

### Study design

A research study with a qualitative approach^(18)^. As already indicated, it was anchored in the ecological model for understanding violence, proposed by the WHO. This study followed the Consolidated Criteria Guidelines for Qualitative Research Reports - COREQ^(19)^.

### Study locus and participants

The study was carried out in a municipality of the Central Region of the state of São Paulo, Brazil, which has 221,950 inhabitants according to the 2010 census, with an estimative of 249,415 inhabitants for 2018.

A school in a peripheral neighborhood was selected, characterized as a group exposed to high social vulnerability for an urban sector, being classified as category 5 (five) according to the 2010 São Paulo Social Vulnerability Index (*Índice Paulista de Vulnerabilidade Social*, IPVS)^(14)^.

The study participants were 8^th^ and 9^th^ grade students, regularly enrolled and attending the selected school. All those who voluntarily agreed to be included in the study participated, signing the Assent Form and the Free and Informed Consent Form by the responsible person. If over 18, a FICF was signed. The choice for this period is justified because it is a time to experiment with intimate relationships^(20)^. The choice for inclusion by teaching period (8^th^ and 9^th^ grade) and not by age group was consistent with the ecological concept of understanding/acting in the face of violence proposed by the WHO.

During the study period, there were 140 students enrolled; of these, 34 chose to participate in the study. Seven students who did not attend during data collection were excluded; as well as two who were transferred to another school, and six that did not bring the terms signed. Despite not participating in the research, these students were heard through participation in an extension project.

### Procedures for data collection

Focus groups and the field diary were used as data collection instruments. To characterize the participants, a questionnaire on socioeconomic characterization and intimate relationships was used.

The focus group is an important strategy for research studies that seek to understand group experiences and transform reality^(21)^. The focus groups had the last author as a moderator; the first author as an observer, and the second author as a rapporteur. It is clarified that the group of researchers participate in the school life through a university extension project, facilitating approach to the adolescents.

The students were invited to this voluntary participation; the following guiding questions were used: How are intimate relationships between adolescents? Which behaviors are positive and which are detrimental in these relationships?

Two focus groups were held on 06/17/2019 and 06/19/2019 with 10 biologically female adolescents, who were invited in the 8^th^ and 9^th^ grade classes; an attempt was made to maintain an equitable number among the classes. The first group had the objective of becoming familiar with the study, with presentation of the participants and discussion of the first triggering question. The second group started with an analysis of the first group to validate the participants› understanding; later, the second question was raised, with deepening of the presence of elements related to TDV. The group was held in a private room at the school, organized in a circle with tables and chairs. At the beginning, name badges were made with the names of each member, so that everyone in the circle recognized each other. Most of the girls were participative during the groups, which lasted a mean of 1 hour, 7 minutes and 55 seconds.

Another two focus groups were held on 10/25/2019 and 10/29/2019 with 09 biologically male adolescents who were invited; an attempt was also made to maintain an equitable number among the classes. Operationalization took place in the same way as the first groups, with a mean duration of 52 minutes and 7 seconds. The participants were identified with the letters PF for biologically female participants (“*Participante Feminino*” in Portuguese) and PM for biologically male participants (“*Participante Masculino*” in Portuguese), and they were numbered based on the sequence in which their speeches appeared in the group.

The groups were recorded using a voice recording application on two cell phones, arranged throughout the room; the recordings were later transcribed in full for analysis. It was decided to carry out separate groups based on biological sexes as indicated by the literature; it is reported that younger adolescents may feel embarrassed to bring experiences in front of participants of different genders^(20)^. The field diary represented a relevant instrument for data analysis and methodological rigor. The investigative experiences were described in it; as well as methodological appropriation; movements, doubts and concerns; and reactions of the participants. In this sense, the reports found in the field diary were incorporated into the transcripts of the groups, constituting the *corpus* for analysis and supporting the inferences made.

In this study, it was decided to seek meaning saturation, which corresponds to a deeper discussion, rich in details and complex with the data to ensure understanding of a phenomenon of interest^(22)^.

### Data analysis

**Figure 1:**
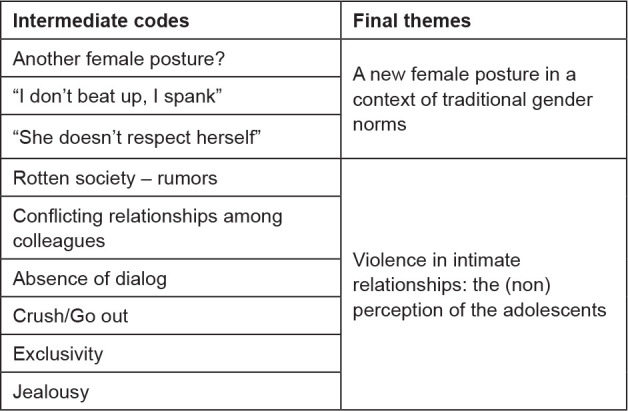
Theme search and review process. 2020

The participants’ characterization was presented by means of descriptive statistics. The qualitative data were analyzed using the reflexive thematic analysis technique^(23)^. Essentially, thematic analysis is a method to identify and analyze patterns of qualitative data. The following steps were conducted for the analysis: (I) familiarization with the data; (II) coding; (III) search by themes; (IV) review of themes; (V) definition and naming of the themes; and (VI) final writing. Stages III and IV can be seen in Figure 1.

To ensure greater data reliability, the transcripts of the groups were returned to the participating adolescents one week after the group was held, for verification, so that they could indicate whether they agreed or wanted to add something to the group construction. It was carried out in an individual printed report, with a blank space for additions or deepening of questions; no adolescent added content to the reports. The construction of initial codes, and of intermediate and final themes took place between two independent researchers, and a third one was resorted to for conflict resolution.

### Ethical aspects

The study followed the recommendations set forth in Resolutions No. 466/2012 and 510/2016 on research involving human beings, being initiated only after approval by the Committee of Ethics in Research with Human Beings. The directors of the selected school were consulted about research authorization in advance.

## Results

The focus groups were conducted with 19 participants, 09 of which were boys and 10, girls. The mean age of these participants was 14.27 years old, ranging between 13 and 15 years old; 63.1% declared themselves Evangelical; 68.4% of the participants lived with their mother and siblings and 47.3% also lived with their father. With regard to housing, 52.6% lived with 4 to 6 people, and 63.1% lived in a house with 4 to 6 rooms. Regarding the guardians’ schooling, 63.1% reported having completed high school.

In relation to the intimate relationships established, 68.4% (n=13) reported having already dated or gone out with people of the other biological sex, and five adolescents reported going out with or dating people of the same biological sex. Most of the adolescents (47.3%) started intimate relationships when aged between 11 and 12 years old, and 47.3% already started sexual relations with partners of the other biological sex. The adolescents reported few arguments with the person with whom they currently have a relationship (47.3%); 21% reported being victims of violence, one for physical violence and three for psychological; and 36.8% reported themselves as perpetrators of violence, two of them physical violence and five psychological violence.

### Theme 1 - A new female posture in a context of traditional gender norms

In this category, a “new female posture” was discussed, as indicated by the adolescents, and the meanings for each gender identity, which are quite particular and reveal the maintenance of traditional gender norms with adolescents in social vulnerability. The adolescents showed to assume a socially validated posture as male, which is to catch, to approach, to have the initiative:

*The boys approach more, but they’re more afraid and are more snobbish (PF4).*

*Not always, the boys don’t have much posture or attitude, I think it’s women who approach more (PF7).*

*Men have to be lynched, we have to live only in women (PF7).*

Aversion to men, expressed directly in the last speech, emerges related to this new posture. A break in the aggressor-victim relationship is revealed, in a society in which women are often victims and men are aggressors:

*I beat up (PF9).*

*I don’t beat up, I spank (PF3).*

From the point of view of biologically male adolescents, this other female posture is brought up with a pejorative and prejudiced content; they are resistant to acceptance of decision-making or initiative in relationships by people of the female biological sex:

*And there are some, I’m going to tell you the truth, dirty (PM3).*

*Ah, throw the ass in the face (PM2).*

*There are some that we don’t even need to do anything, they already do it for you (PM2).*

*Keeps dancing in front of the others (PM3).*

Having an attitude, that is, breaking with the passive female logic causes astonishment and strangeness in the boys, since it is not a socially accepted behavior on the female posture. This posture, especially identified for those adolescents who date, runs through these understandings - that girls must put themselves in an inferior and submissive position:

*But I’m going to tell the truth here at this school there are some girls who don’t even respect themselves (PM2).*

*We must have the posture, and she must have her posture, put herself in her place, that she seduces (PM1).*

The adolescents also brought up questions related to the clothes worn by the girls, very articulated to their judgment; still they put them in the place of a possession, who always need to be accompanied by the partners:

*Or she goes out with shorts like that, I don’t know what, without being with you (PM3).*

*Ah, when I walk with her I walk right behind her, so that no one can go looking (PM3).*

It is noticed that the female bodies cannot dance, they cannot show themselves, they need to “put themselves in their place”. There is a place that is for them (boys) and another for them (girls), marking the differences. The woman remains in a private place, only finding a way out through the social networks; punctuated the space of the boys as public and externalized, even signaled by parents and/or guardians:

*Ah, it’s because the boy goes out more to play ball, the girl doesn’t, she’s only here on the cell phone (PM2).*

*My mother says... oh sit like a girl... that playing ball is for boys... we feel wrong (PF4).*

### Theme 2 - Violence in intimate relationships: the (non)perception of the adolescents

In this category, the discussion was on how intimate relationships among adolescents are established and the elements in them that lead to the establishment of violent relationships. The adolescents brought up the term *crush* to identify situations of attraction to another person, without the need for establishing a close or even concrete physical relationship:

*It means that he’s handsome, and that I want to be with him, that’s it (PF3).*

*So, it could be you saw a girl, and you think she’s beautiful, only you never had a chat with her, you never talked, you don’t know about her conversations, but you thought she was beautiful, and you found her a crush for you, then you start to like her (PM5).*

From this definition, the relationship can effectively occur or not. In case of an effective relationship, the adolescents report “going out” as a form of quick relationship, characterized by the absence of commitments and agreements:

*I go out with the boy, then I see him on the street and actually pretend I don’t even see* (PF7).

*Yeah, it was just that day (PM3).*

With progression of the relationships, dating can arise, a relationship that is characterized by exclusivity and greater commitment. At this moment, the existence of TDV clearly emerges, characterized by the control and need to abandon other activities that are not carried out with the partner:

*Ah, dating, you’ll always have her there by your side* (PM2).

*Yeah, you’ll be faithful* (PM3).

*I don’t know, you can’t go dancing* (PM3).

*Then, it’s about cutting everything too... Because there are many men too that don’t let you, right (PM3).*

*They want to dominate women* (PM1).

Commitment allows control of what the other does and where they goes, and in women this is stronger - especially due to the sense of ownership. The agreements made at the beginning of the relationships go through unhealthy issues, such as non-privacy, distancing or non-maintenance of friends of different genders. These issues are exemplified from the virtual world:

*Yes, when I started dating the agreement was, I only accept a boy on my Facebook and she only accepts a girl on hers, that’s all! About talking, only if it’s a friend, well-known, these things (PM1).*

*I trust, because in addition to everything I have the password for her things (PM2).*

*Yes, when we started dating we agreed, you pass on your password, and I pass on mine (PM2).*

*She leaves her cell phone unlocked, I go there and watch (PM3).*

In this sense, certain difficulty was perceived that these adolescents have to discern intimate, private, and public issues. There is existence based on dependence of the other:

*I stay on the phone all the time, I always want to know what he’s doing* (PF9).

*And also madam, there are people who do fake just to watch the boyfriend, right? [The group agrees]* (PF5).

There is evidence of the control of the other understood as the result of this serious and trustful relationship but, in reality, the relationship is abusive. Jealousy is present in the relationships and contributes to the increase in violent actions in them. The adolescents showed that jealousy is part of the relationships and is sometimes considered as a form of affection in relationships.

*Jealousy is normal people... (PF9).*

*No, there’s no jealousy you know? It’s fear of losing the person* (PM7).

*It’s because without jealousy you can’t manage it* (PM3).

*Yeah, jealousy is a bigger thing, you like that person, that person just wants you* (PM2).

*Quarrel starts from jealousy* (PM1).

Jealousy emerges as a trigger of violence, but there is just no direct perception of it. The statements about jealousy are commonly linked to the social networks. These situations appear again related to the other’s feeling of possession and control:

*I once broke the cell phone (PF8).*

*What if a girl comments on the Facebook photo then?* (PF10).

*I like the comment* (PF7).

*I already curse the girl (PF9).*

*[About constant partner surveillance on Facebook] You’re just leaving a warning, hey, he’s yours!* (PF3).

Throughout the meetings with the adolescents, a dispute between them was noticed in heterosexual relationships. When they felt jealous, they generally turned against the possible “traitor” girl. When discussing the importance of trust for building healthy relationships, the adolescents unveiled that it must be tested and proven, maintaining a look that goes through unhealthy issues:

*Ah, confidence is built up, right, madam! (PM1).*

*You have to prove that you are reliable (PM3).*

*With your posture, right? Madam! (PM1).*

*Yeah, and in the beginning you always have to be suspicious indeed (PM8).*

## Discussion

The findings of this study allowed concluding that the meanings of being a man and a woman have a direct implication in the establishment of violent intimate relationships among adolescents in social vulnerability. A female posture that seeks to break with socially constructed stereotypes emerged, even if in violent and unhealthy ways. A perspective still plaster cast, mainly by the boys, differentiates the postures of being a man and a woman, including the delimitation of different places to be occupied. TDV appears more clearly in dating relationships, in which commitment allows for control and possession. Jealousy is inherent in the relationships and in trust-building, triggering violent actions veiled by the adolescents; the social networks are transversal to these relationships. In this aspect, the dialogic of jealousy is perceived as love and quarreling, as trust and violence.

Considering the above, the discussion about gender within the scope of TDV is inevitable; this question runs transversally in this study. The expectations of the roles of being a man and being a woman in the Brazilian society, present at the social level but with direct consequences for the others, are reinforced by the adolescents. Traditional gender norms endorse beliefs that men must be in a dominant social position that gives them privileges and power over women^(24)^.

A discussion recognized in the literature is the intergenerational transmission of these norms. A number of studies have addressed this discussion about the reproduction of a culture that favors inequality between genders and, consequently, situations of violence^(6,25)^. One study examined the intergenerational influence on the quality of the intimate relationships among adolescents based on their mothers’ experiences. In it, the adolescents apprehend dynamics from observing their mothers, leading to the intergenerational continuity of healthy or violent relationships. This theory postulates the repetition of behavior patterns that the adolescents observed in their parents’ relationships^(26)^. Looking at the families of these adolescents can contribute to the advancement of this knowledge and to the understanding of how gender differences are built in the family dynamics.

The diversity herein found is being highlighted, that is: Brazilian adolescents who live in a highly vulnerable urban region. International studies have addressed these differences in understanding and coping that can advance knowledge of TDV^(25,27)^; in Brazil, the discussion is still incipient^(20)^. A literature review with meta-analysis identified that higher rates of victimization and perpetration of physical TDV were found in samples from neighborhoods with fewer resources (weakened social ties, lower social control, fewer economic opportunities) and with a higher percentage of ethnic minorities for girls^(25)^.

A study of community relationships and TDV experimentation processes among adolescents and young individuals in the United States^(27)^ revealed that adolescents who live in neighborhoods whose residents intervene in or discourage violent behaviors are less likely to experience TDV. In the sense of social cohesion, the study discussed that neighborhoods in disadvantaged conditions tend to weaken or limit social ties, reducing the possibility of the individual seeking help or using social resources to prevent violence. Less favored areas can be more exposed to TDV risk factors, and may see violence as an expected, tolerated or necessary response^(27)^.

The adolescents bring a movement of freedom and search to overcome the stereotypes of femininity. Most of these aspects occur in unhealthy ways, either in the aversion and rejection of the boys, as well as through violence against them. The developmental perspective related to gender and TDV has been highlighted in the literature. Intimate relationships usually start in adolescence, and the adolescents’ knowledge of these relationships is derived from the media and from observing friends and family members. This process is permeated by narcissism, an attachment to specific gender roles and mystification of romantic love, leaving this population especially vulnerable to TDV^(28)^.

This perspective is used in a meta-analysis to determine the prevalence of physical and sexual TDV, as well as its associated factors^(25,29)^. In the emergence of hetero-affective dating relationships, female adolescents tend to be more physically aggressive towards boys at the beginning of these relationships^(29)^. One explanation can be the childhood legacy when it comes to problem solving; girls tend to be more aggressive and boys are socially trained to inhibit these behaviors. Aggression against boys by girls is also less socially questioned than aggression by boys against girls. However, as the female adolescents gain skills in intimate interactions and in the progress of relationships, this situation tends to be altered with reinforcement of the social gender norms already discussed^(25)^. In any case, it is important to point out that the literature has pointed out greater consequences for girls, including inversion of this relation throughout the relationship^(29)^. Another point that deserves to be highlighted is the possibility of boys underreporting situations of violence given the social stigma of violence by boys against girls, or even for them to accept themselves as victims of violence^(25)^.

Furthermore, this finding evidences the importance of studies that address younger adolescents and the construction of intimate relationships in this population. Other studies have essentially addressed older adolescents, with relationships and violence being constructed in different ways. In these studies, the adolescents report higher frequencies of TDV and greater relevance for the perpetration of violence by boys^(6,30)^.

The progression of violence in intimate relationships seems to be associated with the progression of commitment in relationships. The term *crush* is used for a relationship still idealized, which has not materialized; going out remains as a physical approach, which can evolve into a dating relationship. These concepts are reinforced by another study; except the term *crush*, which for younger adolescents emerges in a place still idealized in the relationship^(31)^. It is at the time of courtship that violence gains lighter and invisible shades by the adolescents. In such relationship, dominance, possession, non-privacy, and distancing from friends are allowed; despite the major influence of gender stereotypes; these aspects are present reciprocally in the relationships.

Jealousy is inherent in adolescent relationships, understood as proof of love; it is linked to the trust-building movement cited by the adolescents. It ends up being the great propagator of the different types of violence. In this sense of tolerance of violent behaviors in the relationships between adolescents, it is noticed that there is naturalization of violence, especially considering jealousy as a demonstration of love. Possibly, such acceptance is justified in family origins, through aggressive experiences among the parents, or even in the difference between gender roles when thinking about male dominance in the relationships, which is socially acceptable^(32)^. A qualitative Brazilian study developed with adolescents revealed the occurrence of jealousy related to socially shared beliefs in idealized relationships. Control was closely associated with it, as well as the feeling of possessing the other^(33)^. It is implicit violence that causes other types of it.

Jealousy and the consequent TDV take on new shapes through the use of technologies; such aspect is relevant when considering the ecological model at its relational level, since the digital social networks are transversal to the way of being and relating in the world of contemporary adolescence and its relationships. Constant control and monitoring are supported by the digital resources; a number of studies have denoted this role of technologies in the outline of TDV in Brazil^(1,33)^ and in the world^(4,34)^. A qualitative study with adolescents aged from 12 to 18 years old, carried out in the United Kingdom, sought to explore the role of using technologies in their intimate relationships^(4)^. Unlike our findings, this study found that girls exercise more control and monitoring through the social networks than boys. In order to understand this movement, the authors discussed the relationships of attachment and insecurity^(35)^. In intimate relationships, there may be a need for responsiveness from the partner and for a total sense of security^(4)^. Due to the adolescent developmental process, especially in the fact of experiencing relationships and their identity with peers, this fact acquires special relevance. In addition, aspects related to intimacy, publicity and privacy are emerging in discussions about intimate relationships with adolescents, and should be part of programs and actions that foster healthy relationships and prevent violence^(4,33)^.

International studies have shown the importance of developing preventive actions and programs for peers and spectators within the scope of TDV. These programs have significantly reduced perpetration and victimization by TDV^(36-37)^. In the Brazilian context, it may be interesting to develop actions that promote solidarity among girls, in view of the findings mentioned in this paper. A study conducted in schools in Pennsylvania, United States, found that the students were excited about discussing healthy and unhealthy relationships with school nurses. Many adolescents involved in TDV reported to these nurses to inform their experiences^(38)^.

The limitations were related to the non-deepening of the intergenerational transmission of gender norms among the adolescents. In addition, the focus groups were carried out by dividing the participants by biological sexes; although this configuration allows for the emergence of some content, the interaction between boys and girls could bring new knowledge. The use of individual interviews could also complement the data.

Finally, despite the limitations, the study has important implications for practice, training, and research in adolescents’ health, namely: (1) looking at a population and theme still neglected in health actions; (2) elements that can be linked to strategies and programs to promote healthy relationships and primary prevention of TDV; (3) the need for early interventions with adolescents due to the presence of social rules that are the precursors of violence, with a focus on actions that promote gender equality.

The comprehensive-interpretive process of the data allowed for the addition of scientific advances in the area of adolescent health, as it presents findings for understanding the TDV phenomenon, giving visibility to this problem and thus contributing to the prevention of violence between intimate partners in adult life. It is expected that, despite the uniqueness of the results, the categories developed may, from the elements identified, contribute knowledge applicable in other contexts, in order to enable the implementation of initiatives in Nursing care and assistance for this population.

## Conclusion

Resuming our initial objective, this study allowed accessing the perception of adolescents on TDV. It was revealed that traditional gender norms still occupy a significant place in the outline of violent relationships among adolescents. Such violence is more visible in dating relationships, when commitment and exclusivity are seen as the main characteristics, authorizing possession and control. These instances of violence are veiled and understood as necessary to build trust. Jealousy emerges as the main trigger for violence, and the technologies appear as contemporary resources to reinforce TDV.

The originality and relevance of this study are reiterated: the participation of adolescents who are younger than those frequently included in these surveys, belonging to regions of high social vulnerability and in a developing country.

Further studies on the theme are needed to deepen the understanding of transmission and coping with gender norms among children and adolescents. In addition, the perceptions of families, schools, and the community regarding TDV can contribute with new elements for the design of ecological actions to face the phenomenon.
